# BMP2 gene delivery to bone mesenchymal stem cell by chitosan-g-PEI nonviral vector

**DOI:** 10.1186/s11671-015-0906-3

**Published:** 2015-04-29

**Authors:** Jianhui Yue, Jun Wu, Di Liu, Xiaoli Zhao, William W Lu

**Affiliations:** Center for Human Tissues and Organs Degeneration, Institute of Biomedicine and Biotechnology, Shenzhen Institutes of Advanced Technology, Chinese Academy of Sciences, 1068 Xueyuan Rd., Shenzhen, 518055 People’s Republic of China; Department of Orthopaedic and Traumatology, The University of Hong Kong, 21 Sassoon Rd., Pokfulam, Hong Kong, 999077 People’s Republic of China; Department of Pharmacology, Harbin Medical University, 157 Baojian Rd., Harbin, 150081 People’s Republic of China; Shenzhen Key Laboratory of Marine Biomedical Materials, Shenzhen Institutes of Advanced Technology, Chinese Academy of Sciences, 1068 Xueyuan Rd., Shenzhen, 518055 People’s Republic of China

**Keywords:** Chitosan, Polyethylenimine, Stem cell, Osteogenic differentiation, Gene delivery

## Abstract

Nanotechnology has made a significant impact on the development of nanomedicine. Nonviral vectors have been attracting more attention for the advantage of biosafety in gene delivery. Polyethylenimine (PEI)-conjugated chitosan (chitosan-g-PEI) emerged as a promising nonviral vector and has been demonstrated in many tumor cells. However, there is a lack of study focused on the behavior of this vector in stem cells which hold great potential in regenerative medicine. Therefore, in this study, *in vitro* gene delivering effect of chitosan-g-PEI was investigated in bone marrow stem cells. pIRES2-ZsGreen1-hBMP2 dual expression plasmid containing both the *ZsGreen1* GFP reporter gene and the BMP2 functional gene was constructed for monitoring the transgene expression level. Chitosan-g-PEI-mediated gene transfer showed 17.2% of transfection efficiency and more than 80% of cell viability in stem cells. These values were higher than that of PEI. The expression of the delivered BMP2 gene in stem cells enhanced the osteogenic differentiation. These results demonstrated that chitosan-g-PEI is capable of applying in delivering gene to stem cells and providing potential applications in stem cell-based gene therapy.

## Background

Bone regeneration is one of the major focus points in the field of regenerative medicine. Bone morphogenetic protein-2 (BMP2) is a well-known stimulus for bone regeneration [1]. Currently, nanotechnology has made a significant impact on the development of drug delivery system including the gene delivery [2]. This procedure addresses some of the shortcomings associated with BMP2 protein treatment such as the short half-life and extremely high cost [3,4]. Gene therapy could not only reduce the cost of the treatment but also prolong the release of BMP2 at the regeneration site to coincide with bone formation [5,6].

The success of gene therapy depends on the development of the efficient and safe gene delivery system. Viral vectors are the preferred system in clinical trials due to their high transfection efficiency; however, the safety concerns in terms of immune response and insertional mutagenesis make the nonviral vectors more and more attractive [7-9]. Nonviral vectors show the advantages in easy manufacturing, low immune response, as well as the unrestricted genetic material carrying capacity [9,10]. In clinical, the transient gene expression property of nonviral vector can avoid the adverse effect of BMPs’ overexpression [11].

Among various nonviral vectors, cationic polymer has emerged as a promising one for gene therapy. The structural flexibility of polymer allows for the variety of modification to fabricate a more powerful vector in terms of efficacy and multifunction [12]. Chitosan, known for its excellent biocompatibility and biodegradability, is one of the most widely used cationic nonviral vectors [13]. However, its low transfection efficiency limits its application. There is an increasing interest in improving chitosan’s properties by various modifications. Polyethylenimine (PEI) is another promising cationic nonviral vector and has been proven as one of the most powerful and versatile members of nonviral vector both *in vitro* and *in vivo* [14,15]. However, this vector possesses relatively high cytotoxicity with dose and molecular weight dependency [16]. It has therefore not yet been used in human studies.

In recent years, many pilot studies had proven that the combination of chitosan and PEI can simultaneously enhance the transfection efficiency and decrease the cytotoxicity [17-19]. This formula could be further improved with the properties of targeted delivery [20-23], prolonged *in vivo* circulation [20], and stimuli-responsive [24] by specific structure modification. However, most of these studies were carried out in tumor cells such as HeLa [20,24-26], HepG2 [27], and A549 cells [28], or targeted for tumor treatment [21,29-31]. There are only a few studies left using it to deliver gene to somatic cell such as murine macrophage cells [22] and osteoarthritis [32]. In our previous study, the bioreducible low molecular weight PEI-conjugated chitosan (chitosan-g-PEI) was developed, characterized, and applied to deliver gene to osteoblast cells [33]. It was also simply tried in stem cells. However, there is a lack of a detailed study focused on the behavior of this vector in stem cells, which is very important in the regenerative medicine.

Vectors usually show the cell type-dependent transfection properties because of the differences in cell cycle, cell division frequency, endocytic capacity, and metabolic activity [34]. Mesenchymal stem cells (MSCs) are usually more difficult to transfect [35]. In recent years, the investigation of MSCs and their clinical application have attracted extensive interests. Some nonviral vectors have demonstrated their efficiency in delivering BMP2 gene to MSC such as liposome and PEI [36,37]. So far, there are few nonviral vectors that have been applied in stem cells, leaving very limited choices for stem cell-based gene therapy. Therefore, chitosan-g-PEI should be expected to show its effect on stem cells.

In this study, chitosan-g-PEI was evaluated on delivering BMP2 gene to bone marrow stem cells and compared with chitosan and PEI in terms of the transfection properties and the transgene function *in vitro*. pIRES2-ZsGreen1-hBMP2 dual expression plasmid containing both ZsGreen1 GFP reporter gene and BMP2 functional gene was fabricated to monitor the transgene expression level. The BMP2 expression in the transfected stem cells was measured by the enzyme-linked immunosorbent assay (ELISA) and Western blot. Alkaline phosphatase (ALP) activity and Alizarin Red S staining were performed to determine the osteogenic differentiation and matrix mineralization.

## Methods

### Materials

Chitosan (MW = 10 kDa) with a 92% degree of deacetylation was supplied by AK Biotech Ltd. (Jinan, Shandong, China). Polyethylenimine (PEI,MW = 1.8 and 25 kDa) and 3-[4,5-dimethylthiazol-2-yl]-2,5-diphenyl tetrazolium bromide (MTT) were purchased from Sigma-Aldrich (Shanghai, China). N-Succinimidyl 3-(2-pyridyldithio)-propionate (SPDP) was provided by Thermo Scientific (Rockford, LA, USA). PEI 25 kDa was purified by dialysis in deionized water and lyophilization before used as positive control for gene delivery. Alizarin Red S, cetylpyridinium chloride (CPC), and alkaline phosphatase (ALP) yellow liquid substrate were obtained from Sigma. The BCA™ protein assay kit was obtained from Thermo.

### Construction of pIRES2-ZsGreen1-hBMP2 dual expression vector

Plasmids pCI-neo-hBMP2 and pIRES2-ZsGreen1 (Clontech, Mountain View, CA, USA) were maintained in the lab. ZsGreen1 as GFP homolog is a bright green fluorescent protein derived from a reef coral and modified for higher solubility, brighter emission, and rapid chromophore maturation. Plasmid pIRES2-ZsGreen1-hBMP2 was prepared by inserting hBMP2 cDNA into pIRES2-ZsGreen1 plasmid at Xho I and BamH I sites using restriction enzyme (TaKaRa, Otsu, Shiga, Japan) and T4 DNA ligase (NEW ENGLAND BioLabs, Ipswich, MA, USA). hBMP2 cDNA was amplified by PCR with the forward primer 5′- CCGctcgagACCATGGTGGCCGGGACCCGCT-3′ and the reverse primer 5′ -CGCggatccCTAGCGACACCCACAACCCTCCA-3′ by KOD-Plus-Neo DNA polymerase (TOYOBO, Osaka, Japan). Then, the product was transformed to competent DH5α for amplification. Positive clones were picked for mini plasmid preparation (QIAGEN, Hilden, Germany). The recombinant plasmid pIRES2-ZsGreen1-hBMP2 was characterized by PCR, restriction enzyme digestion, and sequencing analysis, respectively. For cell transfection, the plasmid was purified by Pure Yield™ Plasmid Midiprep System (Promega, Madison, WI, USA) and then examined by gel electrophoresis and NanoDrop ultraviolet spectrophotometer.

### DNA complexes preparation

Chitosan-g-PEI was prepared according to the previous study using SPDP heterobifunctional crosslinking reagent to conjugate chitosan (10 kDa) with PEI (1.8 kDa) [33]. Briefly, SPDP was firstly reacted with the amino group of chitosan and PEI, respectively, to prepare thiolated polymers. Then, chitosan-g-PEI was prepared by oxidization of reduced thiols on PEI and chitosan at room temperature in air to form a disulfide linkage. The feed ratio of chitosan to PEI was 0.25:1 (*w*:*w*), in which formula, it showed the optimal transfection properties in our previous study [33]. Chitosan was purified and dissolved in 50 mM NaAc/HAc buffer (pH5.4). PEI 25 kDa was dissolved in deionized water.

The DNA complexes were prepared freshly by adding the equal volume of sterilized polymer solution to DNA stock. The mixtures were briefly vortexed and incubated at room temperature for 30 min for complexes formation. The complexes of different vectors for gene transfer were prepared at their optimal formula as 20:1, 20:1, and 2:1 for chitosan, chitosan-g-PEI and PEI, respectively. Various weight ratios of the complexes were prepared by manipulating the concentration of polymer solutions. The critical complex ratio was determined by gel electrophoresis.

### Complexes characterization

The DNA condensation ability was investigated by gel retardation. Complex solutions contained 0.1 μg of DNA with various weight ratios that were loaded into gels running at 90 V for 20 min. The particle size and surface charge of complexes were measured by Zetasizer Nano ZS instrument (Malvern Instruments, Malvern, Worcestershire, UK) in triplicate at 25 °C. The complexes containing 2 μg of DNA at various weight ratios (0.5:1 to 25:1) were diluted by NaCl solution to 1 mL. The size was presented as the average value of five runs. The morphology of the complexes was observed by transmission electron microscopy (TEM; Philips Tecnai G2 20 S-TEM, Hillsboro, OR, USA). One drop of each complex solution was carefully dropped on a clean copper grid and negatively stained by 1.5 wt% phosphotungstic acid (pH 6.7). The samples were dried at room temperature before imaging.

### Cell culture

Murine bone marrow cells were collected by flushing the bone marrow cavities of 6-week-old wild-type male C57BL/6 mice euthanized by cervical dislocation and then purified by its physical propensity of adherence to plastic flasks [38]. The animal experiment and care were approved by the Institution Animal Care and Use Committee (IACUC) of Shenzhen Institutes of Advanced Technology, Chinese Academy of Sciences. Cells were cultured in minimum essential medium (alpha) (α-MEM, Mediatech, Herndon, VA, USA) supplemented with penicillin (Sigma-Aldrich), streptomycin sulfate (Sigma-Aldrich), and 20% fetal bovine serum (FBS, Atlanta Biologicals, Lawrenceville, GA, USA) at 37 °C in a 5% CO_2_ humidified incubator. After 72 h of adhesion, nonadherent cells were removed and adherent cells were cultured an additional 7 days with a single media change. Cells were purified by flask adherence through several passages. African green monkey kidney cells (COS-1) were cultured in Dulbecco’s modified Eagle medium (DMEM,Mediatech) supplemented with 10% fetal bovine serum (FBS,Atlanta Biologicals). This cell was used for confirmation of the construction of pIRES2-ZsGreen1-hBMP2 plasmid.

### *In vitro* differentiation of BMSC into multilineage cells

To assess the multilineage differentiation capacity, the obtained bone marrow stem cells (BMSC) underwent osteogenic, adipogenic, and chondrogenic induction by different culture media.

For osteogenic differentiation, cells were cultured with osteogenic medium with α-MEM supplemented with 10% FBS, 10^−7^ M dexamethasone (Sigma-Aldrich), 10 mM β-glycerol phosphate (Sigma-Aldrich), and 50 mM ascorbate-2-phosphate (Sigma-Aldrich). After 3 weeks of differentiation, the mineralization was stained by Alizarin Red S staining. For adipogenic differentiation, cells were cultured with α-MEM supplemented with 10% FBS, 10^−6^ M dexamethasone, 0.5 μM isobutylmethylxanthine (IBMX, Sigma-Aldrich), and 10 ng/mL of insulin (Sigma-Aldrich) for 2 weeks. Lipid accumulation was identified by Oil Red O staining. For chondrogenic differentiation, cells (1 × 10^6^) were seeded in polypropylene tubes with DMEM supplemented with 10^−7^ M dexamethasone, 1% insulin-transferrin-selenium (ITS, Sigma-Aldrich), 50 μM ascorbate-2-phosphate, 1 mM sodium pyruvate (Sigma-Aldrich), 50 μg/mL of proline (Sigma-Aldrich), and 20 ng/mL of TGF-β3 (R&D Systems, Minneapolis, MN, USA). After 3 weeks in culture, the pellets were fixed in 10% buffered formalin for 48 h and embedded in paraffin. Then, 4 μm thick sections were processed for toluidine blue staining (Sigma-Aldrich).

### Transfection efficiency and cytotoxicity

The transfection efficiency was investigated by flow cytometry. Cells were seeded in 6-well plates at an initial density of 4 × 10^5^ cell well^−1^ and allowed to reach 70% to 80% confluence. Before transfection, cells were washed with PBS and refreshed with antibiotic-free medium. Then, the cells were treated with complexes containing 4 μg of pIRES2-ZsGreen1-hBMP2 plasmid and incubated for 24 h. Chitosan (10 kDa) and PEI (25 kDa) were used for comparison in gene delivery. Untreated cell was used as the negative control. Before examination, cells were refreshed with complete medium and cultured for another 24 h. The *ZsGreen1* GFP expression was observed under a fluorescence microscope and quantified by flow cytometry.

*In vitro* stem cell viability under transfection condition was evaluated by MTT assay. Cells were seeded in 96-well plates at an initial density of 1× 10^4^ cells well^−1^ and cultured in a 100-μL medium containing the complexes. The procedure was similar to the course of transfection for evaluating their cytotoxicity during transfection. The MTT assay was performed according to the manufacturer’s instruction, and the results were shown as the percentage of cell viability comparing with the control group. Each value was averaged from six independent experiments.

### BMP2 expression

BMP2 gene was delivered to BMSC. After transfection, the supernatant was collected for testing the BMP2 expression utilizing a commercially available ELISA kit (PeproTech Inc., Rocky Hill, NJ, USA). The activation of its downstream signaling pathway was investigated by Western blot analysis. Cells were lysed in immunoprecipitation buffer (50 mM Tris-HCl pH 7.5, 150 mM NaCl, 1% TritonX-100, 0.5% sodium deoxycholate) containing protease inhibitors. After centrifuge of the lysates, the supernatants were separated by SDS-PAGE and blotted onto a nitrocellulose membrane (Bio-Rad Laboratories, Hercules, CA, USA). The proteins were analyzed with anti-Smad1 and anti-phospho-Smad1/5/8 (Western blot) antibodies, and visualized by SuperSignal West Femto Substrate system (Thermo).

### Osteogenic differentiation

After transfection of BMP2 gene, BMSC were induced for osteogenic differentiation in osteogenic medium. Fourteen days after transfection, the alkaline phosphatase activity (ALP) assay was performed. Cells were rinsed with PBS and lysed in the buffer containing 0.1% (*v*/*v*) Triton X-100, 1 mM MgCl2, and 20 mM Tris. The freezing and thawing process was followed to disrupt the cell membranes. Samples were incubated with ALP substrate solution at 37°C for 30 min and then stopped by 3 M NaOH. These were performed in 96-well microplate with triplicate, and the assay was read against the blank at 405 nm by microplate reader. The total protein content was determined by BCA kit. Calcium deposit was stained by Alizarin Red S staining and quantified at around 21 days. Briefly, cells were washed with PBS and fixed with 10% formalin for 30 min. Alizarin Red S staining working solution with pH value around 4.1 ~ 4.3 was used to stain the cells. Thoroughly washed by distilled water, the calcium deposit with the red color could be observed under a microscope. Quantification of calcium deposition was made by extracting Alizarin Red S staining with 10% cetylpyridinium chloride (CPC) and measuring the absorbance at 570 nm.

### Statistical analysis

All the data presented are expressed as Mean ± SD and the statistical analysis was made using ANOVA and a multiple comparisons test. The difference was considered statistically significant when *p* value was less than 0.05.

## Results and discussion

Chitosan-g-PEI has been demonstrated as a promising gene carrier. In this study, it was applied to bone marrow stem cells in delivering BMP2 gene and compared with chitosan- and PEI-mediated situation. The transfection efficiency, cytotoxicity, and the expression of delivered gene were examined.

### Characterization of the constructed pIRES2-ZsGreen1-hBMP2 plasmid

**Figure 1:**
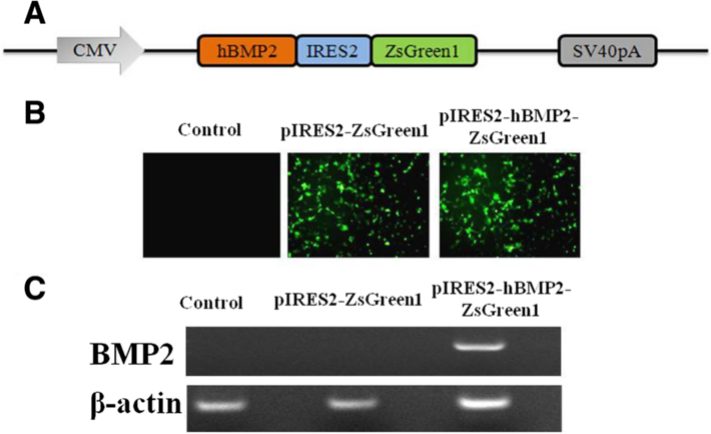
Construction and verification of pIRES2-ZsGreen1-hBMP2 plasmid DNA. pIRES2-ZsGreen1-hBMP2 was constructed containing both the functional gene of BMP2 and the reporter gene of ZxGreen 1 GFP **(A)**. COS-1 cells transfected with pIRES2-ZsGreen1-hBMP2 showed *ZsGreen1* GFP expression observed under a fluorescence microscope **(B)** and the BMP2 mRNA expression by RT-PCR **(C)**.

For conveniently observing the BMP2 expression, pIRES2-ZsGreen1-hBMP2 dual expression plasmid was constructed containing both the functional gene of BMP2 and the reporter gene of *ZsGreen1* GFP as shown in Figure 1A. To some extent, the ZsGreen1 GFP expression could indicate the BMP2 expression level because BMP2 was earlier translated than ZsGreen1 GFP according to this plasmid translation sequence. The IRES element in the plasmid makes sure the encoded proteins expressed separately. Comparing with their fusion protein, this could avoid the adverse interaction in their proteins’ function. The function of this constructed plasmid was examined by ZsGreen1 GFP and BMP2 expression in COS-1 cells. The expressed *ZsGreen1* GFP was observed under fluorescence microscopy as shown in Figure 1B. The insertion of BMP2 gene did not seem to affect the ZsGreen1 GFP expression comparing with the fluorescence images of pIRES2-ZsGreen1 and pIRES2-hBMP2-ZsGreen1. BMP2 expression was examined by its gene expression using real-time reverse transcription PCR (RT-PCR) as shown in Figure 1C. Only pIRES2-hBMP2-ZsGreen1 group showed the positive BMP2 mRNA expression. Both the control group and the pIRES2-ZsGreen1 group showed the negative BMP2 mRNA expression. These results confirmed the successful construction of the plasmid containing both BMP2 and ZsGreen1 GFP for transfection investigation.

### Structure comparison

**Figure 2:**
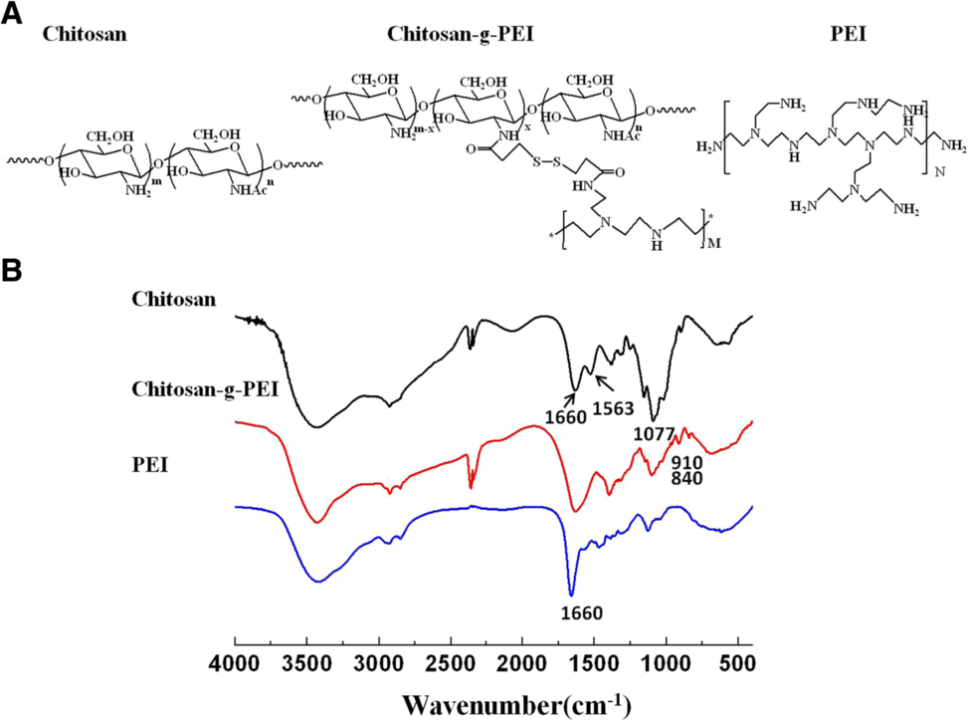
Structure characterization of chitosan-g-PEI by FTIR spectra. The chemical structure of chitosan, chitosan-g-PEI, and PEI was shown **(A)**. Their functional groups correspond to the representative bands on the FTIR spectra **(B)**.

The structure of chitosan-g-PEI, chitosan, and PEI was compared by FTIR spectroscopy as shown in Figure 2. In FTIR spectra, chitosan showed the representative bands at 1,640 cm^−^1, 1,563 cm^−^1, and 1,077 cm^−^1, which were attributed to C = O stretching (amide I band), N-H deformation (amide II band), and C-O stretching vibration. PEI showed the characteristic peaks at 1,660 cm^−^1 for NH2 vibration and 1,139 cm^−^1 for C-N stretching. Chitosan-g-PEI showed the increased signals at 1,660 cm^−^1 corresponding to the NH2 vibration of PEI. At the same time, there were decreased signals at 1,563 cm^−^1 and 1,077 cm^−^1 attributed to the N-H deformation and C-O stretching vibration of chitosan. Meanwhile, the appearance of the signals at 910 cm^−^1 and 840 cm^−^1 corresponding to the C-S stretching vibration were introduced by disulfide linkage in chitosan-g-PEI.

### Characterization of chitosan-g-PEI/DNA complexes

Cationic polymers carrying the positive charge could complex the negatively charged plasmid DNA through electronic interaction to form the nano-scaled particles for cellular internalization and gene expression. The critical complex ratio was investigated by gel electrophoresis. Above the weight ratio of 1:1 (*w*:*w*), the migration of DNA was completely retarded by chitosan-g-PEI.

**Figure 3:**
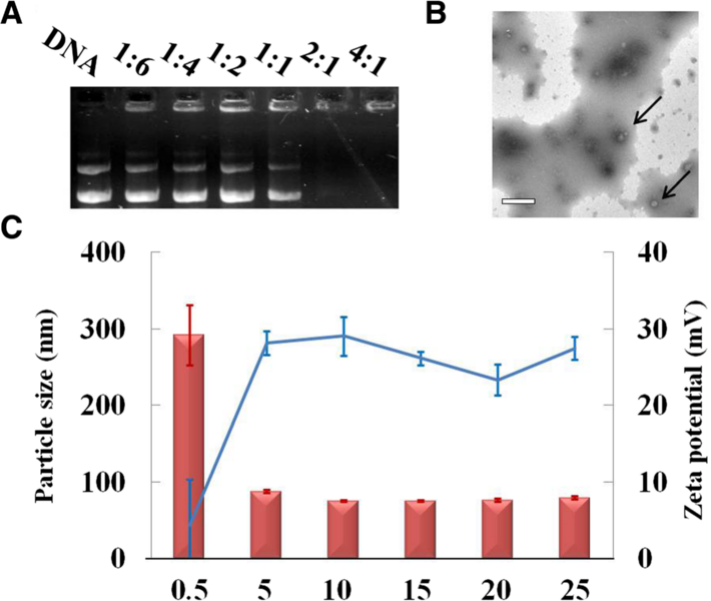
Characterization of chitosan-g-PEI and pIRES2-ZsGreen1-hBMP2 plasmid DNA complexes. The complexes were characterized by the critical complex ration by gel retardation **(A)**, morphology observed by TEM **(B)**, and particle size in histogram and zeta potential in line plot **(C)**. Scale bar: 500 nm.

The formed complexes exhibited as nano-particles with a spherical shape and compacted structure as shown in TEM image (Figure 3B). The particle size and zeta potential of the complexed particles were investigated by static light scattering (SLS) as shown in Figure 3C. Above the weight ratio of 0.5 when the polymers could completely condense DNA, the sizes of the complexes reduced to around 100 nm. At the same time, the zeta potential rose to around 30 mV. Further increasing the weight ratio, the excessive cationic polymer contributed to the surface positive charge, which is necessary for the complexes binding to anionic cell surfaces for cellular internalization.

### Transfection efficiency and cytotoxicity of chitosan-g-PEI on stem cells

**Figure 4:**
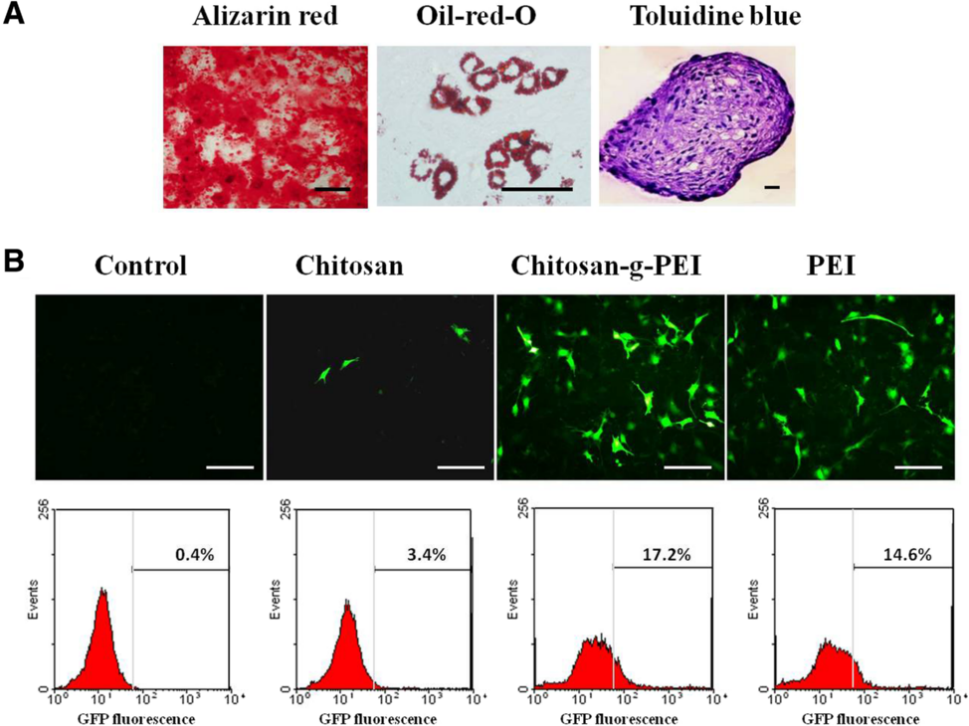
Transfection efficiency of chitosan-g-PEI in bone marrow stem cells (BMSC). The extracted BMSC were capable of conducting the osteogenic, adipogenic, and chondrogenic differentiation by Alizarin Red S, Oil Red O and toluidine blue staining **(A)**. *ZsGreen1* GFP expression in BMSC delivered by gene carriers was visualized by fluorescence and quantified by flow cytometry **(B)**. Scale bar:100 μm.

Mesenchymal stem cells (MSCs) are undifferentiated multipotent cells which reside in various human tissues and have the potential to differentiate into osteoblasts, chondrocytes, adipocytes, fibroblasts, and other tissues of mesenchymal origin [39]. To assess the multilineage differentiation capacity, the extracted bone marrow stem cells underwent osteogenic, adipogenic, and chondrogenic induction by different culture media. It could be observed that these cells are capable of conducting the osteogenic, adipogenic, and chondrogenic differentiation visualized by Alizarin Red S, Oil Red O and toluidine blue staining, respectively (Figure 4A).

pIRES2-ZsGreen1-hBMP2 plasmid was delivered to BMSC by chitosan-g-PEI, chitosan, and PEI. *ZsGreen1* GFP expression could indicate the BMP2-expressed level. The results showed the obvious positive GFP expression in groups of chitosan-g-PEI and PEI as shown in Figure 4B. The chitosan-mediated group showed a low level of GFP expression, and the control group showed the negative results. Analyzed by flow cytometry, the rate of the positive GFP expression cells reached 17.2% and 14.6% for chitosan-g-PEI and PEI, respectively. However, the chitosan-mediated group could only reach 3.4%. All of these efficiencies were actually not very high due to the difficulties in transfecting stem cells [35]. It has been observed that most gene carriers work effectively for immortal cells in culture but fail in transfecting primary cells because of the cell type-dependent differences [40]. It has been reported that PEI showed a much higher transfection level than any other nonviral vector in mammalian cells [41]. Its superior transfection efficiency in most cell types benefits from the ‘proton-sponge’ effect. Chitosan-g-PEI with the component of PEI also got a benefit in delivering BMP2 gene to stem cells.

**Figure 5:**
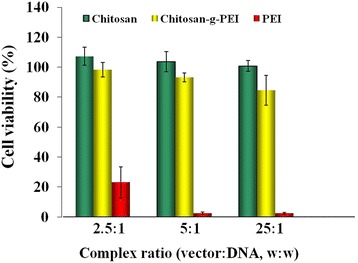
Cell viability of the different complexes in bone marrow stem cells. By MTT assay, chitosan and chitosan-g-PEI showed significant higher cell viability than PEI.

The cytotoxicity of the complexes to BMSC was investigated by MTT assay. Chitosan and chitosan-g-PEI showed significant higher cell viability than PEI as shown in Figure 5. There is no significant cytotoxicity found in the complexes of chitosan. The complexes of chitosan-g-PEI could maintain more than 80% of cell viability when its weight ratio reached 25:1. This result indicated that the selected formula of chitosan-g-PEI complexes with the weight ratio of 20:1 is safe for the stem cells. In the case of PEI, the cells were barely alive above the weight ratio of 5:1. The commonly used formula of PEI complexes weight ratio of 2:1 has already showed very toxic to the stem cells. Studies have found that PEI could induce the rapid perturbation of the plasma membrane with 30 min of exposure. Then, ‘mitochondrially mediated’ apoptotic program was activated over the following 24 h [16]. This toxic was dose-dependent and increased with the molecular weight. Lowering the molecular of PEI could reduce its toxicity. Chitosan-g-PEI with the component of low molecular weight PEI and biocompatible chitosan showed acceptable cell viability in stem cells.

### BMP2 expression

The bone morphogenetic protein (BMP) signaling is required in endochondral ossification and bone formation. BMPs are soluble transmembrane glycoproteins with low molecular weight in normal bone, which belong to the transforming growth factor beta (TGF-β) superfamily [42]. To date, there are more than 20 BMPs members with diverse effects on the growth and differentiation of MSCs, as well as on their ability to synthesize matrix. Among the BMP family, bone morphogenetic protein-2 (BMP2) is a well-known stimulus for bone regeneration and has already been clinically used in the enhancement of the lumbar spine fusion and the treatment of acute tibia fractures. It has been demonstrated with the effect in stimulating the commitment of MSC into osteoblast lineage [43].

**Figure 6:**
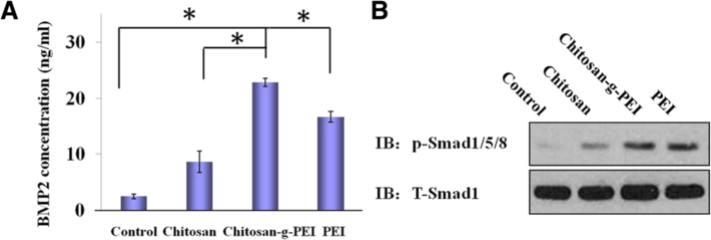
BMP2 expression was examined by ELISA and Western blot assay. The expressed BMP2 protein was quantified by ELISA analysis **(A)**, and the activation of its downstream Smad1/5/8 signaling pathway was studied through Western blot assay **(B)**.

In delivering pIRES2-ZsGreen1-hBMP2 plasmid to BMSC, ZsGreen1 GFP expression could be observed by fluorescence as shown above. The expressed BMP2 protein was investigated by Elisa and Western blot. Three days post-transfection, the secreted BMP2 protein reached around 22 ng/mL in chitosan-g-PEI-mediated group, which was significantly higher than other groups (Figure 6A). The expressed BMP2 functions by activating intracellular Smad proteins. The activated Smad protein complex then regulates the expression of osteoblastic genes by interacting with various transcription factors [44]. By Western blot assay, the activated Smad1/5/8 in the form of phosphorylation was enhanced post-transfection comparing with the control group. The chitosan-g-PEI-mediated transfection showed the significant enhancement in Smad1/5/8 phosphorylation as shown in Figure 6B. The activated Smad complexes were then translocated into nucleus to regulate the transcription of specific target genes. BMP2/Smad signaling is a known mediator of Runx2 expression in starting the osteogenesis [45].

### Osteogenic differentiation of stem cells

Bone formation involves a complex sequence of events including the recruitment, proliferation, and differentiation of stem cell to osteoblast lineage [46]. BMP2 has the effect in stimulating the commitment of MSC into osteoblast lineage and promoting the maturation of osteoblast for bone formation. In bone fracture, MSCs are required to migrate to the injured site and differentiate into osteoblasts to produce the bone matrix for repair [47]. For *ex vivo* MSC transplantation, the genetically modified cells with BMP2 gene showed an enhanced repairing ability in large scaled bone defect [48]. These applications realized based on the effective transfection of MSC by gene carriers.

**Figure 7:**
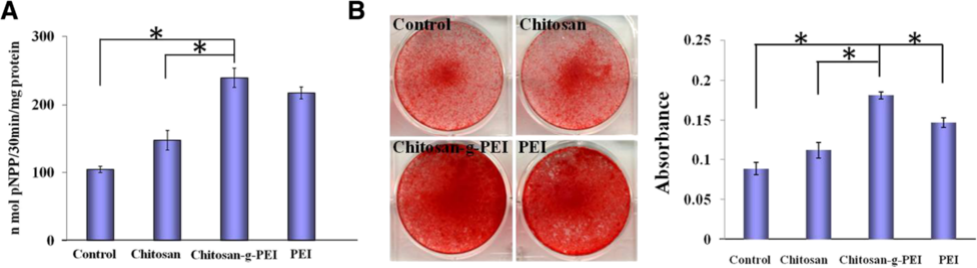
Osteogenic differentiation of the bone marrow stem cells. The osteogenic differentiation was compared by ALP activity **(A)** and mineralization **(B)** between different vector-mediated BMP2 gene transfections.

BMP2 could induce BMSC to differentiate into osteoblast [49]. The osteogenic differentiation was investigated through alkaline phosphatase activity (ALP) and mineralization ability. ALP activity is an important early osteoblastic differentiation marker. Fourteen days post-transfection, the chitosan-g-PEI-mediated group showed highest ALP activities (Figure 7A). PEI showed the slightly lower effect comparing with chitosan-g-PEI, but significant higher than chitosan and control. The similar tendency could be observed in the cells mineralization. Alizarin Red S staining is a common histochemical technique for detecting the calcium deposit in mineralized tissues and cultures. It was conducted on 21 days post-transfection for detecting the cell-mediated deposition of extracellular calcium and phosphate salts. The enhancement of osteogenesis could be observed on chitosan-g-PEI- and PEI-mediated groups (Figure 7B). The quantification of osteogenic differentiation was conducted by extracting the staining using CPC solution to calculate the calcium deposit. Based on these results, chitosan-g-PEI-mediated BMP2 gene transfection showed a significant stronger ability in inducing MSC osteogenic differentiation than the chitosan and control groups, which is even higher than that of PEI. The cytotoxicity of high molecular weight PEI reduced the cell viability leading to the reduced function of expressed transgene.

Bone regeneration utilizing gene therapy either by direct (*in viv*o) or cell-mediated (*ex vivo*) procedure has been extensively studied [50]. Although relatively safer the less efficiency of nonviral vectors compared with viral vectors is still the challenge for their application. Ectopic bone formation could be obtained using adenoviruses and adeno-associated virus (AAV) encoding for several BMP genes by direct intramuscular injection [51-53]. For nonviral vector, gene-activated matrices (GAM) and *ex vivo* procedure are usually used to improve the *in vivo* effect. PEI-mediated BMP4 gene delivery incorporated in a poly (lactic-co-glycolic acid) (PLGA) scaffold could enhance the bone regeneration in a cranial critical-sized defect [54]. In a comparison study, stem cells modified by liposome-mediated BMP2 gene transfer was loaded in collagen sponge for the healing of critical-sized defects in the rat mandible. Although it took slightly longer time than the adenoviral-mediated group, the mature bone matrix could be observed in the central region of the defect 6 weeks after the operation [36]. The *ex vivo* gene therapy for the orthopedic-oriented approaches provides the benefits of cellular component for fast and predictable bone formation. The novel technique of sonoporation and electroporation could further enhance the efficiency of nonviral vector-mediated gene delivery. Stem cell-based BMP2 gene delivery utilizing nucleogection, a method using electroporation for transfection, could induce ectopic bone formation 4 weeks after cell implantation [55].

## Conclusions

Chitosan-g-PEI as nonviral vector was investigated on its *in vitro* gene delivering effect in bone marrow stem cells. pIRES2-ZsGreen1-hBMP2 dual expression plasmid containing both the *ZsGreen1* GFP reporter gene and the BMP2 functional gene was constructed for monitoring the transgene expression level. Chitosan-g-PEI-mediated gene transfer showed 17.2% of transfection efficiency and more than 80% of cell viability in stem cells. These values are higher than the PEI-mediated one. The expression of the delivered BMP2 gene in stem cells enhanced the osteogenic differentiation. These results demonstrated that chitosan-g-PEI is capable of applying in delivering gene to stem cells and providing potential applications in stem cell-based gene therapy.

## Abbreviations

ALP: Alkaline phosphatase
AAV: Adeno-associated virus
BMP2: Bone morphogenetic protein-2
BMSC: Bone marrow stem cells
Chitosan-g-PEI: Low molecular weight PEI-conjugated chitosan
COS-1: African green monkey kidney cells
CPC: Cetylpyridinium chloride
DMEM: Dulbecco’s modified Eagle medium
ELISA: Enzyme-linked immunosorbent assay
FBS: Fetal bovine serum
GFP: Green fluorescent protein
IBMX: Isobutylmethylxanthine
ITS: Insulin-transferrin-selenium
PEI: Polyethylenimine
SPDP: N-succinimidyl 3-(2-pyridyldithio)-propionate
SLS: Static light scattering
TGF-β: Transforming growth factor beta
